# Connectomic neuromodulation for Alzheimer’s disease: A systematic review and meta-analysis of invasive and non-invasive techniques

**DOI:** 10.1038/s41398-022-02246-9

**Published:** 2022-11-21

**Authors:** Cletus Cheyuo, Jurgen Germann, Kazuaki Yamamoto, Artur Vetkas, Aaron Loh, Can Sarica, Vanessa Milano, Ajmal Zemmar, Oliver Flouty, Irene E. Harmsen, Mojgan Hodaie, Suneil K. Kalia, David Tang-Wai, Andres M. Lozano

**Affiliations:** 1 Division of Neurosurgery, Toronto Western Hospital, University Health Network, University of Toronto, Toronto, ON Canada; 2 Krembil Research Institute, Toronto, ON Canada; 3 Functional Neurosurgery Center, Shonan Fujisawa Tokushukai Hospital, Fujisawa, Kanagawa Japan; 4 Neurology Clinic, Department of Neurosurgery, Tartu University Hospital, University of Tartu, Tartu, Estonia; 5 JFK Neuroscience Institute, Edison, NJ USA; 6 Department of Neurosurgery, University of Louisville, School of Medicine, Louisville, KY USA; 7 Department of Neurosurgery, University of South Florida, College of Medicine, Tampa, FL USA; 8 Department of Neurology, Toronto Western Hospital, University Health Network, University of Toronto, Toronto, ON Canada

**Keywords:** Psychiatric disorders, Learning and memory

## Abstract

Deep brain stimulation (DBS) and non-invasive neuromodulation are currently being investigated for treating network dysfunction in Alzheimer’s Disease (AD). However, due to heterogeneity in techniques and targets, the cognitive outcome and brain network connectivity remain unknown. We performed a systematic review, meta-analysis, and normative functional connectivity to determine the cognitive outcome and brain networks of DBS and non-invasive neuromodulation in AD. PubMed, Embase, and Web of Science were searched using three concepts: dementia, brain connectome, and brain stimulation, with filters for English, human studies, and publication dates 1980–2021. Additional records from clinicaltrials.gov were added. Inclusion criteria were AD study with DBS or non-invasive neuromodulation and a cognitive outcome. Exclusion criteria were less than 3-months follow-up, severe dementia, and focused ultrasound intervention. Bias was assessed using Centre for Evidence-Based Medicine levels of evidence. We performed meta-analysis, with subgroup analysis based on type and age at neuromodulation. To determine the patterns of neuromodulation-induced brain network activation, we performed normative functional connectivity using rsfMRI of 1000 healthy subjects. Six studies, with 242 AD patients, met inclusion criteria. On fixed-effect meta-analysis, non-invasive neuromodulation favored baseline, with effect size −0.40(95% [CI], −0.73, −0.06, *p* = 0.02), while that of DBS was 0.11(95% [CI] −0.34, 0.56, *p* = 0.63), in favor of DBS. In patients ≥65 years old, DBS improved cognitive outcome, 0.95(95% [CI] 0.31, 1.58, *p* = 0.004), whereas in patients <65 years old baseline was favored, −0.17(95% [CI] −0.93, 0.58, *p* = 0.65). Functional connectivity regions were in the default mode (DMN), salience (SN), central executive (CEN) networks, and Papez circuit. The subgenual cingulate and anterior limb of internal capsule (ALIC) showed connectivity to all targets of neuromodulation. This meta-analysis provides level II evidence of a difference in response of AD patients to DBS, based on age at intervention. Brain stimulation in AD may modulate DMN, SN, CEN, and Papez circuit, with the subgenual cingulate and ALIC as potential targets.

## Introduction

Dementia affects 55 million people worldwide, with Alzheimer’s disease (AD) making up 70% of cases [1–3]. Ninety-seven percent of patients have late (aged 65 years and older) onset AD (LOAD), with the remaining 3% percent of patients having early (aged less than 65 years) onset AD (EOAD) [4]. LOAD and EOAD have been shown to differ in neuropathology [5–9]. Despite decades of research into pharmacotherapeutic approaches targeting different aspects of the neuropathology of AD [10], no disease-modifying treatment has been established. Recent advances in neuroimaging techniques have revealed the brain networks involved in cognition [11–13]. In patients with AD, resting-state functional magnetic resonance imaging (rsfMRI) connectivity studies have shown dysfunction of these cognitive networks, including the default mode, salience, and limbic networks [14]. Thus, modulation of these dysfunctional brain networks may represent an alternative therapeutic approach to AD.

Invasive and non-invasive techniques of neuromodulation are currently under clinical investigation as brain network-based approaches to treating AD [15]. Deep brain stimulation (DBS), the predominant form of invasive neuromodulation, involves the stereotactic surgical implantation of an electrode in a specific deep subcortical target for controlled, adjustable delivery of electrical pulses for the treatment of various neurological and psychiatric disorders, including AD [16]. While the mechanisms of DBS are not fully understood, it has been shown to either activate or inhibit specific brain networks depending on the site of electrode implantation and stimulation parameters [16]. Non-invasive brain stimulation techniques, predominantly repetitive transcranial magnetic stimulation (rTMS) and transcranial direct current stimulation (tDCS), have also been investigated for brain network modulation in AD [17–19]. The mechanism of rTMS involves the non-invasive, transcranial application of a rapidly changing magnetic field to cause neuronal membrane depolarization, with the generation of action potentials in underlying cortical brain tissue [20]. In tDCS, neuronal membrane modulation and generation of action potentials in cortical brain tissue is achieved via the transcranial application of weak electrical currents (1–2 mA) [21]. While DBS has the disadvantage of being invasive, it targets deep subcortical areas of the brain, which are more difficult to access by non-invasive neuromodulation.

Fox et al. showed an overlap in the brain networks activated by DBS and non-invasive neuromodulation for various neurological and psychiatric diseases [17]. However, the extent of overlap at different hubs within cognitive networks for AD remains unclear, and the overall long-term effect of neuromodulation on cognitive outcome in AD remains unknown. Herein, we performed a systematic review and meta-analysis, and normative functional connectivity analysis to determine the long-term cognitive outcome and patterns of brain network modulation after DBS and non-invasive neuromodulation in AD. The age at AD onset (EOAD and LOAD) determines the age at intervention, which may contribute to therapeutic efficacy. Based on preliminary findings by Lozano et al. that early age at DBS (age <65 years) and late age at DBS (age ≥65 years) differ in therapeutic efficacy [22], we performed subgroup analysis assessing the two subgroups.

## Methods and materials

### Research questions

The objective of this study was two-fold: (1) To assess the combined long-term effect of DBS and non-invasive neuromodulation on cognition in AD, as measured by a normalized cognitive outcome scale, with meta-analysis of the literature, and (2) To determine patterns of overlap in functional connectivity after DBS and non-invasive neuromodulation in AD.

### Literature search and article selection

**Fig. 1:**
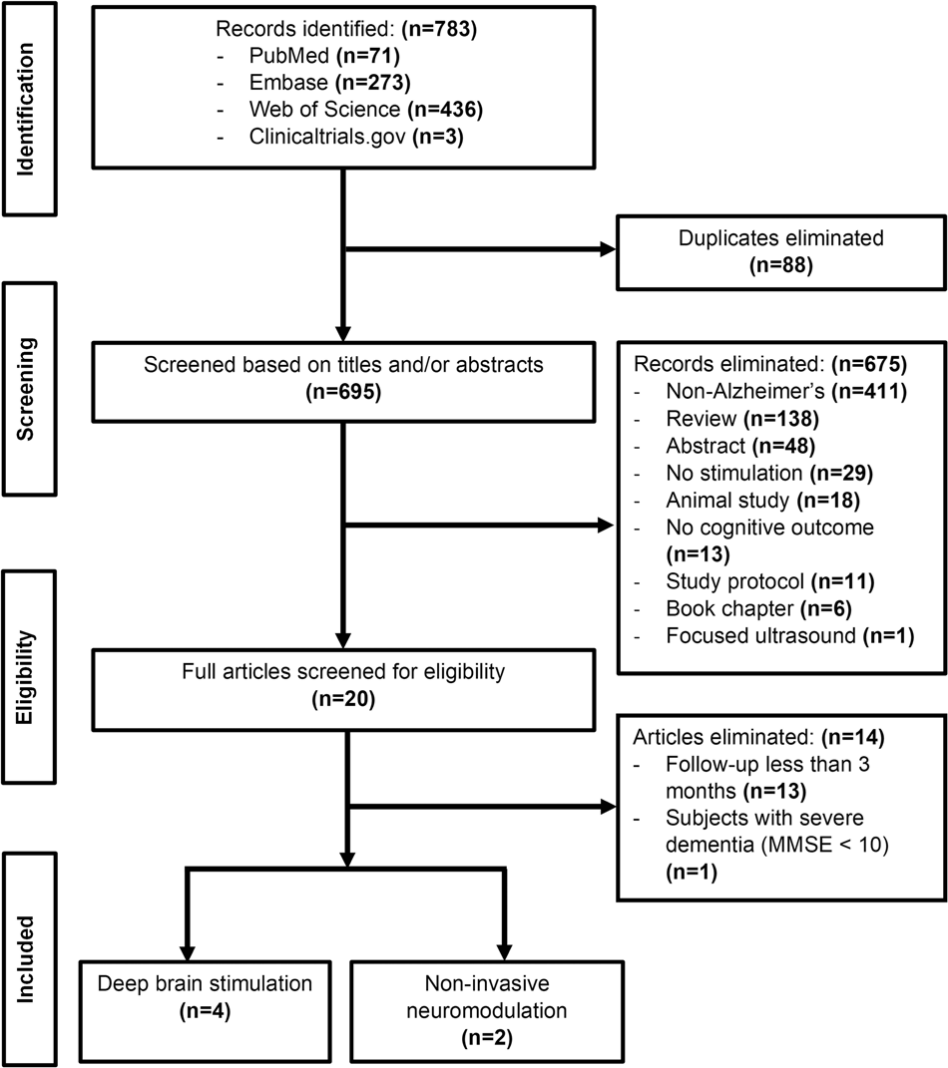
PRISMA flow diagram. MMSE, mini-mental state examination.

We performed a systematic search on PubMed, Embase, and Web of Science, using a combination of three topic-related concepts: dementia, brain connectome, and brain stimulation. The specific search terms associated with each concept include (1) dementia—‘dementia’, ‘Alzheimer’s disease’, ‘cognitive disorder’, and ‘memory disorder’; (2) brain connectome—‘connectome’, ‘connectivity’, ‘structural connectivity’, ‘functional connectivity’, ‘brain network’, and ‘neural network’; (3) brain stimulation—‘electric stimulation therapy’, ‘neuromodulation’, ‘deep brain stimulation’, ‘neurostimulation’, ‘transcranial magnetic stimulation’, and ‘transcranial direct current stimulation’. The three concepts and their associated search terms were then combined using the appropriate Boolean operators for searches on PubMed, Embase, and Web of Science. The search was restricted to human studies published in English between 1980 and 2021 (see detailed search strategy in Supplementary – Search strategy). The registry, clinicaltrials.gov, was also searched for publications directly associated with clinical trials on the topic. The titles and abstracts of the records obtained from the search were screened, and duplicates were eliminated. The remaining records were reviewed to eliminate non-Alzheimer’s disease studies, studies with no assessment of cognitive outcome, studies with no brain stimulation (invasive or non-invasive), reviews, conference abstracts, animal studies, study protocols, and book chapters. Eligible studies were then assessed based on our inclusion and exclusion criteria. Inclusion criteria were Alzheimer’s disease study with DBS or non-invasive neuromodulation as an intervention and at least one cognitive outcome measure. Exclusion criteria were less than 3-months follow-up, patients with severe dementia (MMSE < 10), and focused ultrasound as an intervention. To ensure that articles were compared based on only neuromodulation, we eliminated low-intensity focused ultrasound, for which multiple mechanisms in AD have been reported, including blood–brain barrier opening [23], opening of the glymphatic system [24], clearance of amyloid plaques [25], as well as neuromodulation [26]. DBS programming typically takes 3–6 months to achieve maximum benefit [27]. Therefore, to compare DBS with non-invasive neuromodulation, we defined long-term follow-up as at least 3-months of follow-up. The included studies were then dichotomized into DBS and non-invasive neuromodulation (Fig. 1). The Preferred Reporting Items for Systematic Reviews and Meta-Analyses (PRISMA) guidelines [28] were used for this study (Fig. 1).

### Data abstraction

Included articles were reviewed independently by authors CC and KY. The Centre for Evidence-Based Medicine (CEBM): Levels of Evidence (levels I–V) was used for quality assessment of the studies. Level I represents the lowest risk of bias, and level V represents the highest risk of bias [29]. DBS and non-invasive neuromodulation use different brain targets for stimulation. To maximize statistical power, we combined studies using bilateral DBS at different targets (fornix, nucleus basalis of Meynert (NBM), ventral capsule/ventral striatum (VC/VS), also known as anterior limb of the internal capsule (ALIC)). Similarly, we combined studies using different non-invasive neuromodulation techniques at different targets (left primary motor cortex (M1), premotor area (PMA), supplementary motor area (SMA), dorsolateral prefrontal cortex (DLPFC), and dorsomedial prefrontal cortex (DMPFC)). We also combined different measures of cognitive outcome (Alzheimer’s Disease Assessment Scale-Cognitive Subscale (ADAS-Cog), change in ADAS-Cog-13, Mini-Mental State Exam (MMSE), and Clinical Dementia Rating Scale-Sum of Boxes (CDR-SB)) into a normalized cognitive outcome scale. For each study, we used the scale that was listed as the primary cognitive outcome measure. For studies where the outcome was presented as a graph without a numerical summary estimate, a validated plot digitizing tool (WebPlotDigitizer) [30, 31] was used to extract numerical data for analysis. For connectomic analysis, we performed seed-to-voxel functional connectivity mapping of DBS and non-invasive neuromodulation targets using well-established methods previously described [17, 32].

### Statistical methods and connectomic analysis

#### Cognitive outcome meta-analysis

Cognitive outcome meta-analysis was performed using Review Manager 5.4 software (RevMan 5.4) [33]. We performed the analysis using both fixed-effect and random-effects models with 95% confidence intervals, using the mean and standard error for each outcome measure. A Hedges’ g correction was used to standardize the different cognitive outcome scales as the dependent variable. For cognitive outcome measures where the score increases with worsening AD, the mean values were multiplied by −1. Heterogeneity was estimated using *I*^*2*^, and Z-statistic was used to estimate the overall effect size. The results of the meta-analysis were presented as forest plots. A funnel plot was used to display publication bias. To assess the contribution of type of intervention to methodological heterogeneity, we performed subgroup analysis comparing DBS in AD versus non-invasive neuromodulation in AD. The age at AD onset (EOAD and LOAD) determines the age at intervention, which may contribute to therapeutic efficacy. A previous study suggested that the response of AD patients with DBS could vary as a function of the patient’s age, with older patients (greater than 65 years old) deriving greater benefit than patients under this age [22]. This observation prompted us to examine the results of all studies with this age stratification. We performed subgroup analysis comparing early age at DBS (subjects aged <65 years) versus late age at DBS (subjects aged ≥65 years). In studies where subjects were not grouped by age but the ages and primary outcomes of individual patients were provided, we grouped the patients as early age DBS and late age at DBS accordingly (Supplementary - Table S1).

#### Brain connectomic analysis

We used the approach previously described by Fox et al. [17] to perform normative functional connectivity mapping of DBS and non-invasive neuromodulation targets, utilizing resting-state functional magnetic resonance imaging (rsfMRI) scans of 1000 healthy subjects of the Brain Genomics Superstruct Project (GSP, https://dataverse.harvard.edu/dataverse/GSP) [34]. To define cognitive brain networks, we included in our connectomic analysis studies that employed interventions with known evidence of brain network modulation during active stimulation. We included DBS studies [22, 35–37] since DBS is known to modulate brain networks as part of its therapeutic mechanism [16]. The study by Naro et al. was included since it measured transcranial alternating current stimulation (tACS)-induced changes in gamma band oscillations [38]. Gamma electrical activity refers to electroencephalogram (EEG) oscillations at a frequency of approximately 30–100 Hz in localized central neural pathways, including cognitive brain networks [39]. The study by Li et al. was also included because it measured rTMS-induced evoked potentials as evidence of brain network modulation [40]. Briefly, the seeds of DBS targets (bilateral fornix, NBM, and ALIC) and non-invasive neuromodulation (left DLPFC and left M1, PMA, SMA, DLPFC, and DMPFC) were created from anatomical atlases in standard Montreal Neurological Institute (MNI) space. We used cubic seeds for fornix, NBM, and ALIC that were previously published in Lead-DBS (https://www.lead-dbs.org) [41]. To create the seeds for the study by Naro et al. [38] the 10–10 electroencephalography sensor positions specified in that study (M1 (C3), DLPFC (AF3-AF7), DMPFC (AF3-F1), PMA (FC3), and SMA (FCz)) were first converted into Talairach coordinates as described by Koessler et al. [42]. The Talairach coordinates were then converted to MNI coordinates using an online Talairach to MNI converter with Brodmann Areas (BioImage Suite MNI-TAL) [43]. The MNI coordinates obtained were then used to create graded spherical seeds for left M1, PMA, SMA, DLPFC, and DMPFC from an anatomical atlas, using the method described by Yamamoto et al. [32]. The graded spherical seed of the left DLPFC for the study by Li et al. was created using the MNI coordinates specified in that study (MNIx,y,z = −44,40,29) [40]. The seeds for DBS studies (bilateral fornix, NBM, and ALIC) and non-invasive neuromodulation (left DLPFC and M1, PMA, SMA, DLPFC, and DMPFC) were used to create whole-brain voxelwise 2 × 2 × 2 mm optimal connectivity models (r-maps) using Lead Connectome Mapper software v2.3.2 (https://www.lead-dbs.org) [41]. The r-maps were then converted to t-maps and thresholded by an absolute value of *t* = 5.1, which corrected for multiple comparisons across the entire brain using Bonferroni corrections at a significance level of *p* < *0.05*. The thresholded t-maps were then binarized to obtain meaningful spatial patterns of connectivity associated with DBS and non-invasive neuromodulation. For the study by Naro et al., which used multiple targets (left M1, PMA, SMA, DLPFC, and DMPFC) [38], the binarized maps of the individual targets were combined into one binarized map. The binarized connectivity maps were then summed across DBS and non-invasive neuromodulation targets to reveal how often different brain regions were functionally connected to the target sites. Finally, the binary overlap of the DBS and non-invasive neuromodulation connectivity sum maps was computed to determine areas that shared functional connectivity to both DBS and non-invasive neuromodulation. The overlap represents the number of targets that had a significant time-course correlation in the selected region (Supplementary – Figs. S1 and S2).

### Standard protocol approvals, patient consents, and registrations

This study is a meta-analysis and did not require patient consent or Institutional Review Board (IRB) approval. The study has been registered in PROSPERO, an international prospective register of systematic reviews (registration number: CRD42022307441) [44].

## Results

Our search initially identified 783 records. After the removal of duplicates, 695 studies remained. After that, the screening process (refer to the Methods and Materials section) identified 20 eligible studies. Applying the inclusion and exclusion criteria resulted in the elimination of 14 studies, leaving a total of six included in the meta-analysis. These six studies consisted of four DBS and two non-invasive neuromodulation studies (Fig. 1). For three of the DBS studies [22, 35, 36], each patient served as their own control with cognitive outcome assessed in DBS OFF and DBS ON states. In the study by Scharre et al., three AD patients with DBS were compared with 96 patients from the Alzheimer’s Disease Neuroimaging Initiative (ADNI) [37]. The non-invasive neuromodulation studies involved 110 AD patients. In the study by Li et al., 37 AD patients received rTMS, and 38 AD patients received sham treatment [40]. In the study by Naro et al., 35 AD patients initially had cognitive outcome assessed before receiving tACS [38]. Thirty-two out of the 35 patients were then reassessed for cognitive outcome at the end of the study follow-up. Across all six studies, the total number of AD patients included in the analysis was 242 (36 from DBS studies, 96 from ADNI, and 110 from non-invasive neuromodulation studies). Two studies were classified as level I and four studies as level II on the Oxford Centre for Evidence-Based Medicine (CEBM): Levels of Evidence scale [29]. The included studies are summarized in Table 1. For subgroup analysis, the four DBS studies [22, 35–37] were subdivided into early age at DBS (<65 years) and late age at DBS (≥65 years), as described in the Methods and materials section (Supplementary - Table S1). The two non-invasive neuromodulation studies [38, 40] could not be subdivided by age at intervention because individual patient ages and outcome data were not provided.

**Table 1:** Summary of studies used in meta-analysis.

Author Year	N Stim/No stim	Dx	Stimulation		Connec.	Outcome		F/U (m)	CEBM	Ref
			Type	Target		Measure	Net effect			
Kuhn et al. (2015)	6/6	AD	DBS	Bilateral NBM	FDG-PET, EEG	ADAS-Cog	stable	12	II	[36]
Laxton et al. (2010)	6/6	AD	DBS	Bilateral Fornix	FDG-PET, sLORETA	ADAS-Cog	stable	12	II	[35]
Li et al. (2021)	37/38	AD	rTMS	Left DLPFC	None	ADAS-Cog	stable	3	I	[40]
Lozano et al. (2016)	21/21	AD	DBS	Bilateral Fornix	FDG-PET	Change in ADAS-Cog-13	stable	12	I	[22]
Naro et al. (2016)	32/35	AD	tACS	Left M1, PMA, SMA, DLPFC, DMPFC	EEG	MMSE	stable	24	II	[38]
Scharre et al. (2018)	3/96	AD	DBS	Bilateral VC/VS	FDG-PET	CDR-SB	stable	12	II	[37]

*AD* Alzheimer’s disease, *ADAS-Cog* Alzheimer’s Disease Assessment Scale-Cognitive Subscale, *CDR-SB* Clinical Dementia Rating Scale-Sum of Boxes, *CEBM* Oxford Centre for Evidence-Based Medicine: Levels of Evidence scale; *Connec.* connectomics, *DBS* deep brain stimulation, *DLPFC* dorsolateral prefrontal cortex, *DMPFC* dorsomedial prefrontal cortex, *Dx* diagnosis, *EEG* electroencephalography, *FDG-PET* fluorodeoxyglucose-positron emission tomography, *F/U* follow-up (in months), *M1* primary motor cortex, *MMSE* Mini-Mental State Exam, *NBM* nucleus basalis of Meynert, *PMA* premotor area, *Ref* reference, *Stim* stimulation, *rTMS* repetitive transcranial magnetic stimulation, *sLORETA* standardized low resolution brain electromagnetic tomography, *SMA* supplementary motor area, *tACS* transcranial alternating current treatment, *VC/VS* ventral capsule/ventral striatum.

### Cognitive outcome meta-analysis

**Fig. 2:**
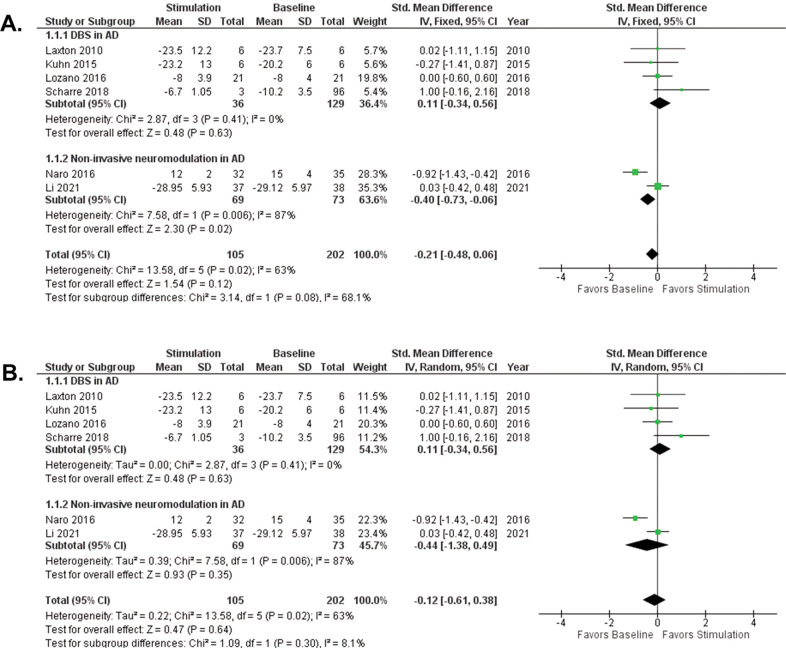
Meta-analysis forest plots. **A** Fixed-effect model of meta-analysis of cognitive outcome after DBS and non-invasive neuromodulation in AD. **B** Random-effects model of meta-analysis of cognitive outcome after DBS and non-invasive neuromodulation in AD. *AD* Alzheimer’s disease, *CI* confidence interval, *IV* inverse variance, *SD* standard deviation.

We first performed a fixed-effect meta-analysis on all AD patients. To account for methodological heterogeneity due to different methods of neuromodulation, we performed subgroup analysis, with subgroups DBS in AD and non-invasive neuromodulation in AD. The fixed-effect model comparing baseline with stimulation (DBS and non-invasive neuromodulation) showed an overall effect size of −0.21(95% confidence interval [CI] −0.48, 0.06, *p* = *0.12*), in favor of baseline. The effect size for DBS in AD was 0.11(95% [CI] −0.34, 0.56, *p* = *0.63*), in favor of DBS. The effect size for non-invasive neuromodulation in AD was −0.40(95% [CI] −0.73, −0.06, *p* = *0.02*), in favor of baseline. The heterogeneity of the DBS in AD subgroup was low (*I*^*2*^ = 0%) compared to the heterogeneity of the subgroup, non-invasive neuromodulation in AD (*I*^*2*^ = 87%). The test for differences between these two methods of intervention was Chi^2^ = 3.14, df = 1 (*p* = *0.08*), *I*^*2*^ = 68.1%, (Fig. 2A). The random-effects model showed an overall effect size of −0.12(95% [CI] −0.61, 0.38, *p* = *0.64*), in favor of baseline. The effect size of DBS in AD was 0.11(95% [CI] −0.34, 0.56, *p* = *0.63*), in favor of DBS. The effect size of non-invasive neuromodulation in AD was −0.44(95% [CI] −1.38, 0.49, *p* = *0.35*), in favor of baseline (Fig. 2B). The fixed-effect and random-effects analyses showed the same effect size for DBS in AD, indicating low methodological heterogeneity within this subgroup. In contrast, the fixed-effect analysis of non-invasive neuromodulation in AD showed an effect size different from that determined by the random-effects model, indicating high methodological heterogeneity within this subgroup.

**Fig. 3:**
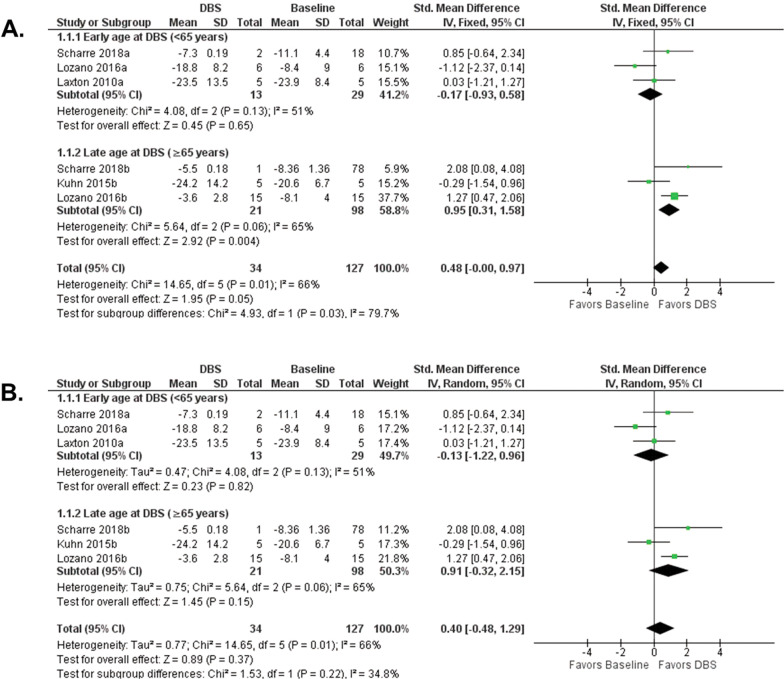
Subgroup analysis forest plots. **A** Fixed-effect model subgroup meta-analysis of cognitive outcome in early age at DBS ( < 65 years old) and late age at DBS ( ≥ 65 years old). **B** Random-effects model subgroup meta-analysis of cognitive outcome in early age at DBS ( < 65 years old) and late age at DBS ( ≥ 65 years old). AD, Alzheimer’s disease; CI, confidence interval; DBS, deep brain stimulation; IV, inverse variance; SD, standard deviation.

Since the neuropathology of EOAD and LOAD patients differ [5–9], and this could affect age at intervention and therapeutic efficacy, we assessed clinical heterogeneity by performing subgroup analysis of early age at DBS (<65 years old) and late age at DBS (≥65 years old). Fixed-effect subgroup analysis showed an overall effect size of 0.48(95% [CI] −0.00, 0.97, *p* = *0.05*), in favor of DBS. The effect size of DBS in AD patients <65 years old was −0.17(95% [CI] −0.93, 0.58, *p* = *0.65*), in favor of baseline. The effect size of DBS in AD patients ≥65 years old was 0.95(95% [CI] 0.31, 1.58, *p* = *0.004*), in favor of DBS. The test for differences based on age at DBS showed Chi^2^ = 4.93, df=1 (*p* = *0.03*), *I*^*2*^ = 79.7% (Fig. 3A). The random-effects model showed an overall effect size of 0.40(95% [CI] −0.48, 1.29, *p* = *0.37*), in favor of DBS. The effect size for DBS in patients <65 years old was −0.13(95% [CI] −1.22, 0.96, *p* = *0.82*), in favor of baseline. The effect size for DBS in patients ≥65 years old was 0.91(95% [CI] −0.32, 2.15, *p* = *0.15*), in favor of DBS, (Fig. 3B). Since our meta-analysis accounted for the sources of methodological and clinical heterogeneity, we believe the fixed-effect model more appropriately represents the effect size of the neuromodulation interventions in AD, rather than the random-effects model, which by principle ignores heterogeneity [45].

**Fig. 4:**
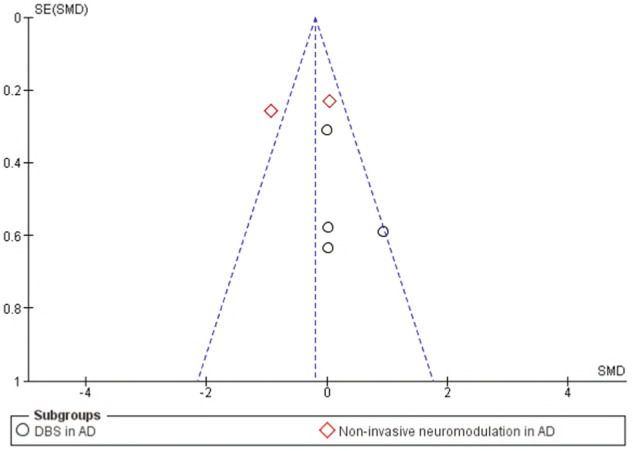
Funnel plot of study data showing publication bias against small negative studies. AD Alzheimer’s disease, DBS deep brain stimulation, SE standard error, SMD standardized mean difference.

A funnel plot was used to assess publication bias. It showed publication bias against small, negative studies in the literature, as indicated by the lack of studies falling on the left lower part of the plot (Fig. 4).

### Brain network mapping analysis

**Fig. 5:**
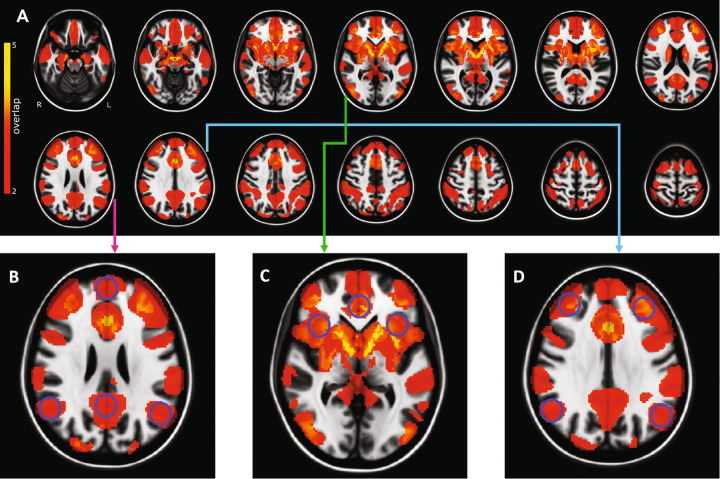
Normative functional connectivity networks for DBS and non-invasive neuromodulation. **A**. Voxels associated with DBS and non-invasive neuromodulation were significantly (*P*_*Bonferroni*_ < *0.05*) correlated with Papez circuit (4 out of 5 (80%) overlap of stimulated targets), salience network (80% overlap), default mode network (40% overlap), and central executive network (60% overlap). The nodes of **B**. Default mode network, **C** Salience network, and **D** Central executive network are indicated by blue circles. L, left; R, right.

Our normative functional connectomic analysis used seeds from DBS and non-invasive neuromodulation targets. The binarized summed functional connectivity maps of these targets, weighted by Hedges standardized mean cognitive outcome, demonstrated significant (*P*_*Bonferroni*_ < *0.05*) time course correlation of blood oxygen level-dependent (BOLD) signals between targets of neuromodulation and regions belonging to the Papez circuit, default mode network (DMN), salience network (SN), and central executive network (CEN) (Fig. 5). The structures of the Papez circuit that showed significant functional connectivity correlation to neuromodulation targets, with an overlap in 4 out of 5 targets (80%), include the anterior thalamus, anterior cingulate, retrosplenial cortex, and hippocampus (Fig. 5A). The salience network structures (dorsal anterior cingulate and anterior insula) also showed significant functional connectivity correlation to neuromodulation targets, with 4/5 overlap (80%) (Fig. 5C). The functional connectivity correlation overlap in DMN was 40% (2 out of 5 targets) (Fig. 5B**)** and 60% in CEN (Fig. 5D). The parts of the brain network that showed the strongest functional connectivity correlation to neuromodulation targets, with 5 out of 5 overlap (100%), were the subgenual cingulate (Supplementary – Fig. S3B) and the anterior limb of the internal capsule (ALIC) (Supplementary - Fig. S3C). There was also 80% overlap in the ventral tegmental area (Supplementary - Fig. S3B).

## Discussion

We performed a systematic review, meta-analysis, and normative functional connectomic analysis of DBS and non-invasive neuromodulation in Alzheimer’s disease. Studies in patients 65 years and older reported improved cognitive outcome with DBS (*p* = *0.004*). On the other hand, DBS in patients younger than 65 years was associated with a lack of improvement in cognitive outcome. Resting state functional MRI (rsfMRI) measures the time course correlation of low-frequency (0.01–0.1 Hz) oscillations in blood oxygen level-dependent (BOLD) signal between brain regions [46], which have been shown to be associated with neuronal activity [47]. We found a significant (*P*_*Bonferroni*_ < *0.05*) time course correlation in BOLD signals between the targets of neuromodulation and regions belonging to DMN, SN, CEN, and Papez circuit, based on a normative connectomic analysis. The subgenual cingulate and the anterior limb of the internal capsule showed the strongest correlation, with 5 out of 5 overlap (100%) in targets that had significant time course correlation to the targets of stimulation. By employing subgroup analysis, we have been able to identify distinct outcome patterns in therapeutic response based on age at intervention (late age at DBS versus early age at DBS), neuromodulation techniques (DBS versus non-invasive), and functional connectivity network correlations. Our findings will guide future study designs in this emerging field.

AD is a complex disease, with LOAD and EOAD exhibiting differences in neuropathology [5–9], functional connectivity [48, 49], and clinical presentation [50]. In a voxel-based morphometric study, using 3 T MRI, Moller et al. found that LOAD patients exhibited atrophy of the hippocampus, right temporal lobe, and cerebellum compared to age-matched controls. In contrast, EOAD patients exhibited atrophy of the hippocampus, bilateral temporal lobes, precuneus, cingulate gyrus, and inferior frontal cortex compared to age-matched controls. A direct comparison between subgroups revealed an increase in atrophy of the precuneus in EOAD and medial temporal lobe in LOAD [6]. Using 18F-fluorodeoxyglucose positron emission tomography, Rabinovici et al. demonstrated that EOAD patients have significantly lower glucose metabolism in the precuneus/posterior cingulate, lateral temporoparietal, and occipital cortices compared to LOAD patients [9]. Patients with EOAD and LOAD also differ in clinical presentation. Mendez et al. showed that 64% of EOAD patients present with non-amnestic symptoms compared to only 12.5% of LOAD patients [50]. These differences dictate the timing of intervention for AD and may affect therapeutic efficacy. Our analysis revealed that the effect of DBS on cognitive outcome differs between patients younger than 65 years and patients 65 years and older. Patients with early-onset (EOAD) and late-onset (LOAD) AD are likely to receive DBS intervention at age <65 years and ≥65 years respectively. The mechanism underlying this response difference based on the age at DBS is unknown. However, since DBS is thought to modulate brain networks in AD, differences in functional connectivity between EOAD and LOAD may partly explain this pattern of response to DBS. In a study comparing functional connectivity of prodromal EOAD and LOAD, Pini et al. demonstrated that prodromal LOAD patients had lower functional connectivity in DMN and limbic networks compared to controls. In contrast, prodromal EOAD patients had lower functional connectivity in frontoparietal (CEN) and visual networks compared to controls [48]. Gour et al. also demonstrated that EOAD exhibited decreased functional connectivity in the dorsolateral prefrontal network and increased functional connectivity in the anterior temporal network compared to controls. The reverse pattern was found in LOAD in the same study [49]. Therefore, considering LOAD versus EOAD in the inclusion/exclusion criteria of future AD deep brain stimulation trials may yield more meaningful results by ensuring that neuropathology-matched groups are compared.

Cognitive processes depend on the integration of complex interactions among large-scale brain networks, including DMN, SN, CEN, and the limbic system, which includes Papez circuit [11, 51–54]. The medial prefrontal-medial parietal DMN is involved in memory and abstract thought [52]. The CEN is comprised of the dorsolateral prefrontal cortex and posterior parietal cortex and is involved in attention, high-level cognitive tasks, and working memory [55]. Functional connectivity studies have shown that cognitively demanding tasks activate the CEN and deactivate the DMN. The cingulo-opercular SN has been shown to serve as the neural switch between CEN and DMN [56]. The Papez circuit is part of the limbic system and is involved in episodic memory and spatial navigation [51]. AD is characterized by the dysfunction of these large-scale neural networks [57]. Our study identified functional connectivity correlation overlaps among networks activated by DBS targets (fornix, ALIC, and NBM) and non-invasive neuromodulation targets (left DLPFC, M1, PMA, SMA, and DMPFC). We found more functional connectivity correlation overlap in SN and Papez circuit (80%) compared to CEN (60%) and DMN (40%). Thus, neuromodulation at these targets in AD may engage CEN and DMN by modulating activity in SN. We also found that the subgenual cingulate and ALIC had 100% functional connectivity correlation overlap to neuromodulation at all stimulation targets. This could mean that neuromodulation at the subgenual cingulate and ALIC may modulate neuronal activity in DMN, CEN, SN, and Papez circuit. While ALIC (also known as VC/VS) DBS for AD [37] and subgenual cingulate DBS for treatment-resistant depression [58] and anorexia nervosa [59] have been performed, to the best of our knowledge, subgenual cingulate DBS has not been performed for AD. This study identifies the subgenual cingulate as a potential target for DBS in AD.

### Limitations of the study

The sample size that met our inclusion criteria was small. We eliminated studies that did not have at least three months of follow-up to ensure that studies were comparable to DBS, which typically requires at least three months of programming to optimize therapy. We found that most non-invasive neuromodulation studies had no follow-up (cognitive outcome was assessed during treatment or at the end of treatment). As a result, we ended up with only two eligible non-invasive neuromodulation studies for comparison with four DBS studies. Although non-invasive neuromodulation appeared not to improve long-term cognitive outcome in AD, the result could be different if there were more non-invasive neuromodulation studies in AD with long-term follow-up. Our study also only covered two non-invasive neuromodulation techniques, rTMS and tACS. The field of non-invasive neuromodulation is rapidly developing, and newer techniques, such as low-intensity focused ultrasound, now exist. However, we eliminated focused ultrasound because multiple mechanisms of low-intensity focused ultrasound in AD have been reported, making it challenging to attribute the therapeutic effect to neuromodulation alone [23–26].

We performed connectomic analysis using normative rsfMRI scans of 1000 healthy subjects of the Brain Genomics Superstruct Project (GSP, https://dataverse.harvard.edu/dataverse/GSP) [34]. Functional connectivity based on rsfMRI has been found to correlate with structural connectivity of white matter pathways determined by diffusion tensor imaging [13]. It is important to recognize that functional connectivity based on rsfMRI of healthy individuals may not reflect the neural network connectivity in AD patients who have neurodegeneration-related anatomical changes. However, studies comparing patient-specific functional connectivity with normative functional connectivity based on atlases have resulted in the identification of the same networks [60, 61]. Thus, our normative functional connectivity analysis may indeed reflect the brain network functional connectivity in AD patients after neuromodulation at the specified targets (fornix, ALIC, NBM, left DLPFC, M1, PMA, SMA, and DMPFC). Another limitation of this study is the inclusion of targets of non-invasive neuromodulation (rTMS and tACS) [38, 40] in the connectomic analysis even though these non-invasive neuromodulation techniques did not appear to have long-term effect on cognitive outcome in this meta-analysis. However, both included both rTMS and tACS studies showed evidence of brain network modulation during short-term active stimulation [38, 40]. Therefore, the combination of DBS and non-invasive neuromodulation targets [22, 35–38, 40] in the connectomic analysis allowed us to define both the subcortical and cortical parts of the neural networks that govern the effects of neuromodulation on cognition. We believe that defining both the cortical and subcortical parts of the cognitive network open the window for innovative therapeutic investigations for AD. It is conceivable that in the future, non-invasive neuromodulation could be used adjunctively to probe the cognitive neural networks before proceeding to invasive neuromodulation.

## Conclusion

Our analysis suggests that AD patients differ in therapeutic response to DBS based on the age at intervention. Disease onset, which determines age at intervention, should be considered in the design of future AD neuromodulation studies. Our normative functional connectivity analysis shows that neuromodulation (invasive and non-invasive) may improve cognition in AD by modulating the triple cognitive networks (DMN, SN, and CEN) and Papez circuit. We found that the subgenual cingulate and ALIC had 100% functional connectivity with all the networks that correlated with neuromodulation. Therefore, the subgenual cingulate and ALIC may be good targets for future DBS trials in AD.

## Supplementary information

Supplementary Figure Legends

Table S1

Figure S1

Figure S2

Figure S3

Search Strategy

## Data Availability

The preprocessed functional connectome and all code and commands necessary to perform the functional connectivity analysis are freely available through Lead-DBS (https://www.lead-dbs.org) [35].
