# Supplementary material for: Connectomic neuromodulation for Alzheimer’s disease: A systematic review and meta-analysis of invasive and non-invasive techniques
Source: Transl Psychiatry. 2022 Nov 21;12:490. doi: 10.1038/s41398-022-02246-9 (PMC9678946; doi:10.1038/s41398-022-02246-9)
Supplement: Supplementary file 1 — Supplementary Figure Legends [file 41398_2022_2246_MOESM1_ESM.docx]

**Supplementary Figure Legends**

**Supplementary - Figure S1:** Seeds used for normative functional connectivity analysis. **A.** Cortical seeds for non-invasive neuromodulation – blue: left DLPFC, red**:** left M1, PMA, SMA, DLPFC, DMPFC, **B.** Nucleus basalis of Meynert (cyan), **C.** Fornix (green), and **D.** Anterior limb of the internal capsule (yellow). L, left; R, right.

**Supplementary - Figure S2:** Approach to identifying normative functional connectivity maps. L, left; R, right; rTMS, repetitive transcranial magnetic stimulation; tACS, transcranial alternating current treatment.

**Supplementary - Figure S3:** Normative functional connectivity networks for DBS and non-invasive neuromodulation. **A.** Voxels associated with DBS and non-invasive neuromodulation were significantly (*P_Bonferroni_ < 0.05*) correlated with Papez circuit (4 out of 5 (80%) overlap of stimulated targets), salience network (80% overlap), default mode network (40% overlap), and central executive network (60% overlap). **B.** Sagittal view showing subgenual cingulate with 100% overlap and ventral tegmental area with 80% overlap. **C.** Axial view showing ALIC with 100% overlap. L, left; R, right.
