# Supplementary material for: Connectomic neuromodulation for Alzheimer’s disease: A systematic review and meta-analysis of invasive and non-invasive techniques
Source: Transl Psychiatry. 2022 Nov 21;12:490. doi: 10.1038/s41398-022-02246-9 (PMC9678946; doi:10.1038/s41398-022-02246-9)
Supplement: Supplementary file 2 — Table S1 [file 41398_2022_2246_MOESM2_ESM.docx]

**Table S1: Subdivision of DBS in AD studies into early age at DBS (<65 years old) and late age at DBS (≥65 years old)**

| **Study**  **Year** | **Age at DBS (years)** | **N**  **DBS/NO DBS** | **Cognitive Outcome*** | | | **Ref** |
| --- | --- | --- | --- | --- | --- | --- |
|  |  |  | **Scale** | **DBS** | **Baseline** |  |
| Kuhn et al., 2015 | a. <65 | 1/1 | ADAS-Cog | 18 | 18 | 36 |
|  | b. ≥65 | 5/5 | ADAS-Cog | 24.2±14.2 | 20.6±6.7 |  |
| Laxton et al., 2010 | a. <65 | 5/5 | ADAS-Cog | 23.5±13.5 | 23.9±8.4 | 35 |
|  | b. ≥65 | 1/1 | ADAS-Cog | 23.3 | 22.8 |  |
| Lozano et al., 2016 | a. <65 | 6/6 | ADAS-Cog-13 change | 18.8±8.2 | 8.4±9 | 22 |
|  | b. ≥65 | 15/15 | ADAS-Cog-13 change | 3.6±2.8 | 8.1±4 |  |
| Scharre et al., 2018 | a. <65 | 2/18 | CDR-SB | 7.3±0.19 | 11.1±4.4 | 37 |
|  | b. ≥65 | 1/78 | CDR-SB | 5.5±0.18 | 8.36±1.36 |  |

AD, Alzheimer’s disease; ADAS-Cog, Alzheimer’s Disease Assessment Scale-Cognitive Subscale; CDR-SB, Clinical Dementia Rating Scale-Sum of Boxes; DBS, deep brain stimulation; Ref, reference; *****mean±standard error
