# Supplementary material for: Connectomic neuromodulation for Alzheimer’s disease: A systematic review and meta-analysis of invasive and non-invasive techniques
Source: Transl Psychiatry. 2022 Nov 21;12:490. doi: 10.1038/s41398-022-02246-9 (PMC9678946; doi:10.1038/s41398-022-02246-9)
Supplement: Supplementary file 6 — Search Strategy [file 41398_2022_2246_MOESM6_ESM.pdf]

STUDY TITLE: CONNECTOMIC NEUROMODULATION FOR ALZHEIMER'S DISEASE: A SYSTEMATIC REVIEW AND META-ANALYSIS OF INVASIVE AND NON-INVASIVE TECHNIQUES

1. PUBMED

Concept 1: Dementia

Search: "Dementia"[Mesh] OR Alzheimer's disease[tw] OR "Cognitive disorder"[tw] OR "Memory disorder"[tw]

Mesh: "Dementia"[Mesh]

Concept 2: Brain connectome

Search: "Connectome"[Mesh] OR Connectivity[tw] OR Connectom*[tw] OR "Structural connectivity"[tw] OR "Functional connectivity"[tw] OR "Brain network"[tw] OR "Neural circuit"[tw]

Mesh: "Connectome"[Mesh]

Concept 3: Brain Stimulation

Search: "Electric Stimulation Therapy"[Mesh] OR Neuromodulation[tw] OR "Deep Brain Stimulation"[tw] OR Neurostimulation[tw] OR "Transcranial magnetic stimulation"[tw] OR "Transcranial direct current stimulation"[tw]

Mesh: "Electric Stimulation Therapy"[Mesh]

FINAL SEARCH: Concepts 1, 2, and 3 combined using Boolean operator AND, with filters Humans, English Language, from 1980/1/1 - 2021/12/18

Last accessed: December 18, 2021

2. EMBASE

Concept 1: Dementia

Search: Dementia OR Alzheimer OR Cognitive disorder OR Memory disorder

Concept 2: Brain connectome

Search: Connectome OR Connectivity OR Structural connectivity OR Functional connectivity OR Brain network OR Neural Circuit

Concept 3: Stimulation

Search: Electric Stimulation Therapy OR Neuromodulation OR Deep Brain Stimulation OR Neurostimulation OR Transcranial magnetic stimulation OR Transcranial direct current stimulation

FINAL SEARCH: Concepts 1, 2, and 3 combined using Boolean operator AND, with filters Human, English Language, year: 1980 – current

Last accessed: December 18, 2021

3. WEB OF SCIENCE: all databases (Interdisciplinary)

Concept 1 Dementia

Search: TS=(Dementia) OR TS=(Alzheimer's disease) OR TS=(Cognitive disorder) OR TS=(Memory disorder)

Concept 2 Brain Connectome

Search: TS=(Connectome) OR TS=(Brain structural connectivity) OR TS=(Functional connectivity) OR TS=(Brain network) OR TS=(Neural Circuit)

Concept 3 Brain Stimulation

Search: TS=(Electric Stimulation Therapy) OR TS=(Neuromodulation) OR TS=(Deep Brain Stimulation) OR TS=(Neurostimulation) OR TS=(Transcranial magnetic stimulation) OR TS=(Transcranial direct current stimulation)

FINAL SEARCH: Concepts 1, 2, and 3 combined using the Boolean operator AND, with the following filters:

Language: English

Document type: Article

Timespan: 1980 - 2021

Human: TS=("population groups" not "animal models") OR (TS=(men or women or patient or female or male or subjects or adult) NOT TS="animal models")

Last accessed: December 18, 2021
